# AI Diffusion Models Generate Realistic Synthetic Dental Radiographs Using a Limited Dataset

**DOI:** 10.3390/jimaging11100356

**Published:** 2025-10-11

**Authors:** Brian Kirkwood, Byeong Yeob Choi, James Bynum, Jose Salinas

**Affiliations:** 1 Organ Support and Automation Technologies, U.S. Army Institute of Surgical Research, 3698 Chambers Pass, Bldg 3611, Ft. Sam Houston, San Antonio, TX 78234, USA; jose.salinas4.civ@health.mil; 2 Department of Population Health Sciences, University of Texas Health San Antonio, 7703 Floyd Curl Drive, San Antonio, TX 78229, USA; choib@uthscsa.edu; 3 Department of Surgery, University of Texas Health San Antonio, 7703 Floyd Curl Drive, San Antonio, TX 78229, USA; bynumj@uthscsa.edu

**Keywords:** dental radiography, artificial intelligence, deep learning, diffusion model, image generation, synthetic data, data processing, human-in-the-loop, judgment

## Abstract

Generative Artificial Intelligence (AI) has the potential to address the limited availability of dental radiographs for the development of Dental AI systems by creating clinically realistic synthetic dental radiographs (SDRs). Evaluation of artificially generated images requires both expert review and objective measures of fidelity. A stepwise approach was used to processing 10,000 dental radiographs. First, a single dentist screened images to determine if specific image selection criterion was met; this identified 225 images. From these, 200 images were randomly selected for training an AI image generation model. Second, 100 images were randomly selected from the previous training dataset and evaluated by four dentists; the expert review identified 57 images that met image selection criteria to refine training for two additional AI models. The three models were used to generate 500 SDRs each and the clinical realism of the SDRs was assessed through expert review. In addition, the SDRs generated by each model were objectively evaluated using quantitative metrics: Fréchet Inception Distance (FID) and Kernel Inception Distance (KID). Evaluation of the SDR by a dentist determined that expert-informed curation improved SDR realism, and refinement of model architecture produced further gains. FID and KID analysis confirmed that expert input and technical refinement improve image fidelity. The convergence of subjective and objective assessments strengthens confidence that the refined model architecture can serve as a foundation for SDR image generation, while highlighting the importance of expert-informed data curation and domain-specific evaluation metrics.

## 1. Introduction

The practice of dentistry is undergoing a profound transformation with the rapid development of artificial intelligence (AI) technology for use in diagnostics, treatment planning, education, and automation. However, development of AI-based technologies are typically dependent on the availability of large datasets that are necessary for training and testing AI algorithms. Additionally, training data should reflect the underlying population domain characteristics necessary to avoid bias of these models. Although there are some medical imaging datasets (including dental radiographs) that are publicly available, relatively small datasets limit the ability to develop AI models with optimum domain-specific performance for clinical applications [1,2]. In such cases, techniques have been utilized to modify and/or enhance existing images with enough differentiation from the original image to allow for an artificial expansion of the training dataset. These techniques alter the image in various ways (cropping, flipping, noise insertion) such that the basic parameters and characteristics of the image are retained while providing the AI model new variants of the image for training to improve image generation and avoid model overfitting [3]. Another approach is to use synthetic data that has been generated from AI models trained to replicate datasets with the same parameters as the original (i.e., real) data used for the AI model development. This approach was shown to improve the performance of AI models for classification or segmentation tasks compared with no augmentation or conventional augmenting techniques [3,4]. Synthetic medical images have many potential applications from generating synthetic contrast enhanced images to training healthcare providers [5]. Two of the most promising use-cases for inclusion of synthetic data are dataset augmentation (balancing or supplementing available data for AI model development) and privacy protection (approaches that enhance privacy by reducing sharing of real data that may include personal identifiers) [1].

In AI dental applications, the demand for synthetic data is rapidly increasing for various use-cases including augmenting training datasets, enhancing image quality, creating realistic material for dental education, and establishing robust image datasets for large-scale computer simulations. Specifically, the use of image generation models for creation of Synthetic Dental Radiographs (SDR) has potential to replicate distributions (shapes and variance) and structure (correlation among the attributes) of real dental radiographs [6,7]. Recently, image generation using diffusion modeling, a cutting-edge class of generative techniques that create synthetic images that are diverse and realistic, has been shown to outperform other generative models like Generative Adversarial Networks (GAN) and Variational Autoencoders (VAE) [8]. SDRs must be clinically indistinguishable from real images if they are to be used for augmenting training datasets in addition to generating clinically realistic defects and restorations. SDRs should have enough population diversity to increase the generalizability of the AI models developed from synthetic data [9,10].

However, evaluation of SDRs to determine clinical relevance and quality requires analysis by expert clinicians using specific clinically relevant criteria as part of the evaluation. Differences in clinical judgment may influence model development and evaluation of model performance [11,12]. Currently, there are no standardized evaluation metrics that incorporate subject-matter experts (SME), such as dentists, for validation of realism of synthetic images or assessment of image generation model performance [13]. A five-point scale was recently described that assigns 1 to highly unrealistic to 5 highly realistic qualities in the evaluation of synthetic panoramic radiographs based on twelve criteria [14]. In addition, image generation model performance is dependent on multiple data processing steps to construct clean, usable and well-labeled images for training data as part of a data processing pipeline [15]. When a single SME is utilized to evaluate or label clinical data there is the risk that differences in clinical judgement among SMEs can lead to unintentional incorporation of subjectiveness into model development [11]. This includes the use of SMEs as part of a set of iterative steps for AI model development and refinement. The objective of this study was to incorporate dentists into the development process of image generation models and evaluate how incremental refinements influenced three models’ ability to generate realistic SDRs. Because expert evaluation alone can introduce subjectivity, additional quantitative approaches were also applied to provide complementary measures of fidelity.

This study combines subjective evaluation by dentists with quantitative distributional metrics, including Fréchet Inception Distance (FID) and Kernel Inception Distance (KID), to assess the fidelity of SDRs. By integrating both clinical and statistical perspectives, we sought to determine how expert-informed data curation and architectural refinement impact the realism and fidelity of diffusion model generation of SDRs.

## 2. Materials and Methods

### 2.1. Dental Radiograph Database

Over 10,000 raw unenhanced dental radiographs in Digital Imaging and Communication in Medicine (DICOM) format from the Defense Health Agency (DHA) (Falls Church, VA, USA) Enterprise Clinical Image Archive (ECIA) were randomly extracted and de-identified prior to transfer to researchers at the U.S. Army Institute of Surgical Research (USAISR) (San Antonio, TX, USA).

### 2.2. Development of Training Dataset for Model Development

The dental radiograph database underwent six manual processing steps to form the training data packages. (1) Each image was converted to Joint Photographic Experts Group (JPEG) file format with an image output grayscale resolution of 976 × 700 pixels. (2) The images were organized into folders based on the method of image capture (intraoral and extraoral). (3) The intraoral images were further organized into subfolders as periapical radiographs or bitewing radiographs. (4) The bitewing radiographs were then reviewed by a single board-certified dentist to label the image as clinically acceptable or not acceptable. (5) The clinically acceptable bitewing radiographs were then organized by location of the image capture (right molar image, right premolar image, left premolar image and left molar image). Clinically acceptable images were defined as radiographs that are diagnostic image quality, essential anatomic coverage for the region of interest and without technical errors. (6) Clinically acceptable bitewing radiographs capturing the right molar region (BWRM) that satisfy the image selection criteria (Table 1) were used for this study. Images were resized to 128 × 128 pixels using the Python programming language version 3.12 [16] with the Python Image Library, Pillow version 10.4.

### 2.3. Clinical Expert Involvement

#### 2.3.1. Expert Panel (EP) of Dentists

An expert panel consisted of four board-certified dentists (EP1, EP2, EP3 and EP4) with at least 15 years of clinical experience and graduate level research training. The expert panel participated in the “Image Selection Criteria Assessment” survey.

#### 2.3.2. Primary Dentist Evaluator

A board-certified dentist (EP0) with 15 years of clinical experience, 7 years of research and development experience, and 3 years developing, testing and validating AI systems. EP0 is the research team member that performed image processing for model development and participated in the survey to re-confirm which images satisfied the image selection criteria. A minimum of a 30 days elapsed between image processing by EP0 and participation in the survey. EP0 completed the survey prior to collection and analysis of the expert panel survey results.

### 2.4. Survey Instrument

A survey instrument titled “Image Selection Criteria Assessment” was used to determine which dental radiographs should be included in the training dataset for model development. The dentists that participated in this study self-selected a unique identifier code (UIC) that was entered at the end of the survey to link survey results to ensure the anonymity of each participant. The UIC enables investigators to conduct future AI interpretability research associated with the images being evaluated by the SMEs. The UIC entered by expert panel members was linked to one of the following, EP1, EP2, EP3 or EP4, for reporting the data collected. The primary dentist evaluator entered EP0. The survey asked the participants the same question for each of 100 real dental radiographs on whether each image satisfies image selection criteria based on their professional opinion. Each participant was given as much time as necessary to assess the image presented and provide a response. The images associated with the survey were randomly selected from a dataset containing images that satisfy image selection criteria that were previously determined by EP0 (Table 1). The order the images were presented during the survey were randomized. The survey instrument was constructed using SurveyMonkey [17].

### 2.5. AI Model Development

#### 2.5.1. Hardware and Software Configuration

The investigators used a combination of Graphic Processing Unit (GPU) enabled computers and the U.S. Army Artificial Intelligence Institute (A2I2) AI cluster for model development, testing, validation, and image generation. Model development was performed using the Python programming language version 3.12 [16] with the PyTorch learning library version 2.6 [18].

#### 2.5.2. Model Architecture and Training Development

**Figure 1:**
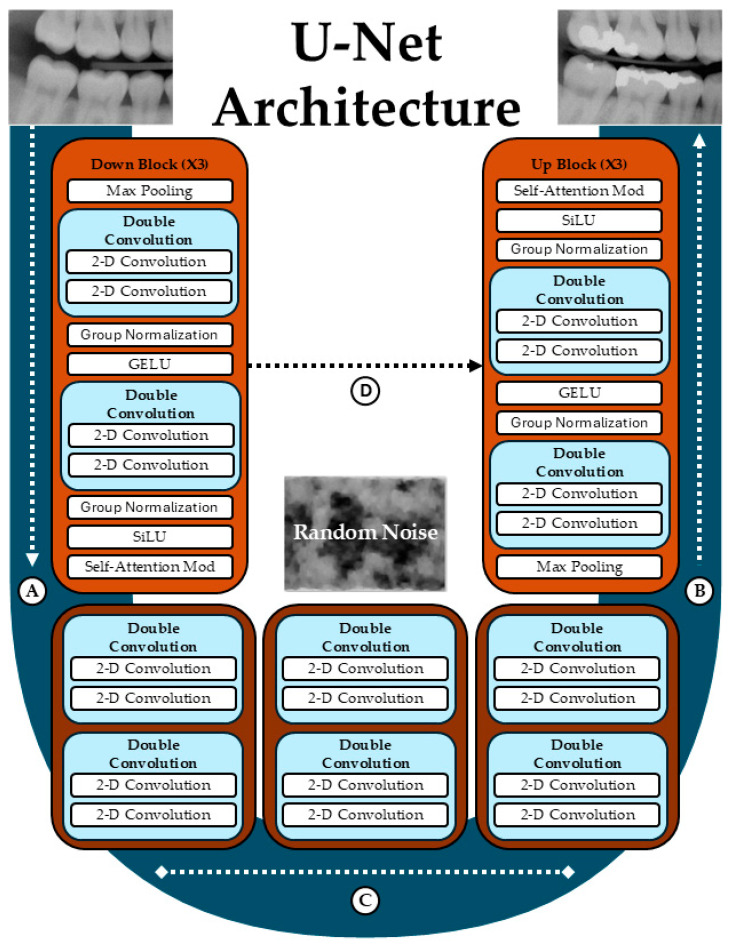
U-Net architecture for diffusion-based image generation. The network consists of a contracting path (**A**), an expansive path (**B**) and a bottom layer (**C**). Skip connections (**D**) link corresponding down and up blocks. Figure is original.

All models were based on a diffusion algorithm using a U-Net architecture with grayscale input and output resolution of 128 × 128 pixel (Figure 1). The U-Net included a contracting path of three down blocks, a set of bottom layers, and a symmetrical expansive path of three up blocks. Each down and up block contained convolutional layers with group normalization and Gaussian Error Linear Unit (GELU) activations, followed by Sigmoid Linear Unit (SiLU) activations and self-attention modules. Skip connections linked corresponding down and up blocks, preserving spatial features during reconstruction.

Diffusion training was implemented by progressively adding Gaussian noise to input images across multiple steps and training the network to reverse this process. This iterative noise–denoise cycle enables the model to generate novel SDRs by starting from random noise. For Models #1 and #2, training employed 300 diffusion steps per epoch, while Model #3 used 600 diffusion steps, representing an architectural refinement designed to improve image fidelity. All models were trained for up to 30,000 epoch using the Adaptive Moment Estimation (ADAM) optimizer with a mean squared error (MSE) loss function. Following training, each model was tasked to generate 500 SDRs for evaluation.

#### 2.5.3. SDR Model #1

Training dataset: Model #1 was trained on 200 BWRM radiographs randomly selected from those judged clinically acceptable by a single dentist and meeting predefined image selection criteria (Table 1). Model development and image generation: Training was performed using this dataset processed through the ECIA pipeline. The model was trained for up to 30,000 epochs, with images generated every 50 epochs for inspection. Realism improved notably around 22,500 epochs, prompting the retention of one version trained to that point. A second version was trained to 40,000 epochs for comparison. Each trained version produced 500 synthetic dental radiographs (SDRs), which were graded using the SDR scoring rubric (Table 2) by a single expert (EP0).

#### 2.5.4. SDR Model #2

Training dataset: Model #2 was trained on a subset of Model #1′s dataset, refined through a multi-step process. 100 images were randomly selected from the 200 images used for training Model #1. The screening dentist (EP0) reconfirmed these images against selection criteria, and then an expert panel of four dentists reviewed the same set. The images identified by the panel that satisfied image selection criteria were used to curate the dataset for model #2 development. To maintain a training set size of 200 images, duplicate images selected by the panel and reconfirmed images from EP0 were included as needed. Model development and image generation: Model #2 was trained for 30,000 epochs using the same diffusion framework as Model #1. After training, the model generated 500 SDRs, which were graded by EP0 using the SDR scoring rubric (Table 2).

#### 2.5.5. SDR Model #3

Training dataset: Model #3 used the same expert-curated dataset assembled for Model #2. Model development and image generation: In contrast to Models #1 and #2, Model #3 incorporated architectural refinement, increasing the number of diffusion steps per epoch from 300 to 600 steps. This modification was intended to enhance image fidelity. The model was trained for 30,000 epochs under otherwise identical conditions. After training, Model #3 generated 500 SDRs, which were graded by EP0 using the SDR scoring rubric (Table 2).

### 2.6. Assessment of Image Generation Model Performance

#### 2.6.1. Expert Evaluation

EP0 assessed and labeled each SDR generated using the SDR scoring rubric (Table 2). Each image was assigned a score equal to the category number (i.e., Category 1 = 1, Category 2 = 2, etc.). Model performance was assessed by comparing each model’s ability to consistently generate realistic SDR. The rate of realistic images generated by a model is used as key performance metric to evaluate and compare model performance. A two-sample proportion test was applied to evaluate if the changes made for each model developed significantly changed the proportion of realistic SDR generated by the models. Statistical analysis was performed using R version 4.3.1 [19].

#### 2.6.2. Distributional Similarity

The similarity between synthetic and real dental radiographs was evaluated using the FID and KID metrics. Features were extracted with a pretrained Inception-v3 network [20]. FID was computed from the Gaussian statistics (mean and covariance) of real and synthetic feature embeddings, while KID was computed as the unbiased squared maximum mean discrepancy (MMD) with a polynomial kernel [21,22]. Because KID approaches zero for samples from the same distribution, small values are expected and relative differences between models are more informative than absolute magnitudes [22]. Lower scores in both metrics indicate greater similarity.

FID and KID were calculated using the 500 SDRs produced by each model. For each evaluation subset of real radiographs (Table 3), N denoted the available images; in each repetition, S ≤ N real images and an equal number of SDRs were sampled. Values of S and the number of repetitions R were set per subset, with means and 95% confidence intervals (CIs) reported across repetitions. Subsets included All200 (N = 200, S = 100, R = 50), Panel57 (N = 57, S = 50, R = 50; seen by all models), and Unseen143 (N = 143, S = 90, R = 50; seen only by Model #1). Additional sets were drawn from Shift1000 (1000 clinically acceptable but training-excluded radiographs). These included the full Shift1000 (N = 1000, S = 150, R = 50), Near40 (40 images closest to the All200 centroid; S = 40, R = 100), and five Random40 subsets (N = 40 each, S = 40, R = 100). A real-vs-real baseline (“floor”) was also estimated for each subset by splitting the *N* real images in half across 200 runs.

## 3. Results

### 3.1. Training Datasets

Out of the 10,000 dental radiographs (raw unenhanced) processed from the ECIA, 2226 BWRM radiographs were identified with 1284 of those labeled as clinically acceptable by EP0 (57.5%). Of the clinically acceptable BWRM radiographs, 225 images satisfied the image selection criteria (Table 4). Of these, a subset of 100 images randomly selected from the Model #1 training dataset (200 images) were incorporated into the survey administered to the expert panel. 79 (43 + 36) out of 100 images all four expert panel members agreed whether the image met or did not meet the image selection criteria. 43 (7 + 36) out of 100 images the majority of the expert panel agreed that the BWRM radiograph satisfied image selection criteria. Only 57 unique BWRM radiographs, each approved for inclusion by at least one dentist, were included in the training datasets for Model #2 and Model #3. (Table 4).

### 3.2. Expert Evaluation of Model Performance

The evaluation of model performance was conducted by a single dentist and all the reported results in this section are based on image analysis by EP0. Grading of the SDRs generated by Model #1 after every 50 epoch from 0 to 30,000 epochs resulted in 466 images out of 4808 images that were labeled Category 1, indicating that 9.7% of the SDRs are realistic images. The average score of the grading of the 4808 SDRs generated was 3.22 with a standard deviation of 1.16. The rolling average score of 72 images generated across 400 epochs was the lowest at 22,750 epochs with an average score of 1.90, The standard deviation of the images scored between 22,550 epochs and 22,950 was 0.95. In addition, the highest percentage (75%) of Category 1 (realistic) images occurred at 22,550 epochs.

Three versions of Model #1 were evaluated by grading 500 images generated by each version. Model #1 trained for 22,500 epochs, generated 28 out of 500 images that were labeled Category 1 indicating that 6% of the SDRs are realistic images. The average score of the grading of the 500 SDRs generated was 3.18 with a standard deviation of 0.98. Model #1 trained for 30,000 epochs, generated 74 out of 500 images that were labeled Category 1 indicating that 15% of the SDRs are realistic images. The average score of the grading of the 500 SDRs generated was 3.09 with a standard deviation of 1.11. Model #1 trained for 40,000 epochs, generated 9 out of 500 images that were labeled Category 1 indicating that 2% of the SDRs are realistic images. The average score following grading of the 500 SDRs generated was 3.83 with a standard deviation of 0.75 (Table 5).

Thirty thousand epochs were selected as the number of epochs to train Model #2 and Model #3 based on the observed performance of the three versions of Model #1. The version trained for 30,000 epochs produced the highest proportion of Category 1 (realistic) images (Table 5). Model #2 generated 110 out of 500 images that were labeled Category 1, indicating that 22% of the SDRs are realistic images. The average score following grading of the 500 SDRs generated by Model #2 is 2.65 with a standard deviation of 1.15. Model #3 generated 451 out of 500 images that were labeled Category 1, indicating that 90% of the SDRs are realistic images. The average score following grading of the 500 SDRs generated by Model #3 is 1.12 with a standard deviation of <0.01. The proportion of realistic SDRs significantly increased when increasing the training from 22,500 epochs to 30,000 epochs while training the model for 40,000 epochs resulted in a significantly less proportion of realistic images than both 22,500 epochs and 40,000 epochs (Table 5). The two modifications during iterative model development from Model #1 to Model #2 and Model #2 to Model #3 were significant (*p* < 0.05) changes resulting in a higher proportion of realistic SDRs (Table 5). There was no significant difference between the average score of the grading of the 500 SDRs generated by the three models.

### 3.3. Objective Analysis of Model Performance

#### 3.3.1. Absolute Performance

Across all evaluation subsets, Model #3 consistently produced the lowest FID and KID values, indicating superior alignment with real radiographs compared to Models #1 and #2 (Table 6). For example, on the All200 set (N = 200, S = 100, R = 50), Model #3 achieved a mean FID of 114.4 (95% CI: 108.5–120.2) and KID of 0.00239 (0.00215–0.00266). By contrast, Model #2 scored 182.2 (176.0–189.3) for FID and 0.00361 (0.00338–0.00389) for KID, while Model #1 scored 221.1 (206.9–235.0) and 0.00374 (0.00342–0.00404), respectively. A similar pattern was observed across the Panel57, Unseen143, Shift1000, Near40, and Random40 subsets.

Model #2 generally outperformed Model #1, especially in FID, although differences in KID between the two were less consistent. For example, on the Panel57 subset (N = 57, S = 50, R = 50), Model #2 achieved an FID of 195.6 (182.9–204.8) compared with 233.6 (218.9–250.4) for Model #1, while their KID values overlapped considerably [0.00370 (0.00332–0.00405) vs. 0.00385 (0.00350–0.00422)]. Model #1 consistently showed the highest FID and KID values across all subsets, indicating the poorest performance.

The lowest real-vs-real “floor” FID mean was the Shift1000 subset at 24.5. The real-vs-real “floor” FID mean for the subset seen by all three models (Panel57) was 80.0. The real-vs-real “floor” FID mean for the subset seen by Model #1 but not by Model #2 and Model #3 (Unseen143) was 50.6. Model #3 FID mean were 126.2 (Shift1000), 127.3 (Panel57) and 117.3 (Unseen143). The ΔFID mean of FID [Model #3] mean minus FID [real-vs-real “floor”] mean are 101.7 (Shift1000), 47.3 (Panel57) and 66.7 (Unseen143). The overall ΔFID mean for all comparison subsets between Model #3 and real-vs-real “floor” was 54.3.

#### 3.3.2. Pairwise Comparisons

Pairwise Δ analyses confirmed these trends (Table 7). Model #3 significantly outperformed both Model #1 and Model #2 across all subsets in FID (all Δ95% CI excluded 0). In KID, Model #3 also significantly outperformed the other models in nearly all subsets, with exceptions limited to smaller subsets such as Random40_2 where ΔKID intervals overlapped 0. Model #2 significantly outperformed Model #1 in FID across all subsets, but KID differences were frequently nonsignificant. At no point did Model #1 outperform either of the other models.

#### 3.3.3. Overall Patterns

The rank ordering of performance was stable across all conditions (Table 8): Model #3 best, Model #2 intermediate, Model #1 worst. This pattern held true for both in-distribution subsets (All200, Panel57, Unseen143) and out-of-distribution generalization tests (Shift1000, Near40, Random40). Real-vs-real “floor” analyses were calculated for each subset, providing a lower bound for achievable scores, and all synthetic models remained above this threshold.

## 4. Discussion

**Figure 2:**
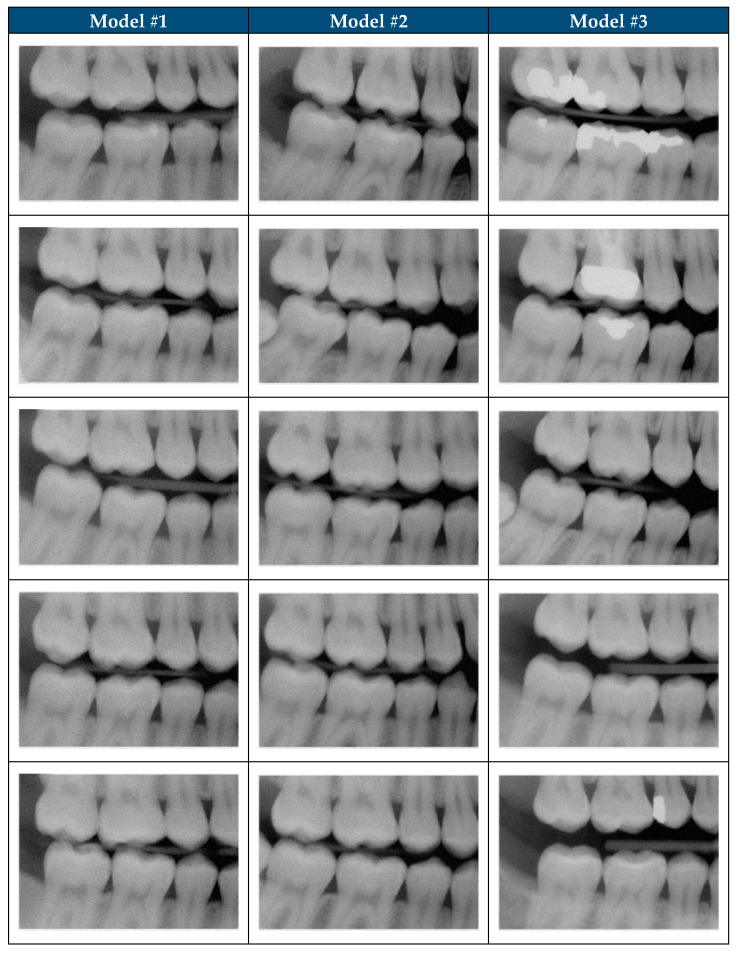
Realistic synthetic dental radiographs generated by the three diffusion models.

Diffusion models have the potential to outperform other generative models like GANs and VAEs in various natural image synthesis tasks [23]. GANs in particular are more prone to mode collapse limiting the diversity of the images [24]. Originally, this project intended to use a GAN model with an internally developed architecture trained with 672 clinically acceptable dental bitewing radiograph for 30,000 epochs. However, when EP0 evaluated 100 SDRs generated by the GAN model; none of the images were realistic and the model experienced mode collapse (unpublished). The design of this study is based on the lessons learned from the GAN model development effort. The models developed to generate SDRs in this study provides empirical evidence of diffusion model effectiveness in a clinically relevant and domain-specific to dental radiographs. Diffusion models, with their stable training dynamics and capacity to generate diverse and high-fidelity images, appear particularly well-suited for capturing the complex features of medical images [23,25,26]. This study demonstrates the potential of diffusion models to generate realistic SDRs (Figure 2), even when trained on a constrained dataset.

The curation process followed a stepwise approach. First, a single dentist screened 10,000 radiographs to determine clinical acceptability then applied predefined image selection criteria, producing a dataset of 200 images used to train the initial model. Second, from this dataset, 100 images were randomly selected and reviewed by an expert panel of four board-certified dentists, that identified 57 different dental radiographs that met image selection criteria. These 57 images were then used to refine the training sets for Models #2 and #3. The results demonstrate that Model #3 can produce realistic SDRs at least 90% of the time based on expert review (Table 5). The substantial leap in the rate of realistic SDR by Model #3 was achieved by incorporating additional denoising steps in the algorithm, which highlights the importance of algorithm optimization.

The 300 additional denoising steps likely allowed the model to better capture the subtle textural nuances and anatomical details inherent in dental radiographs [27,28]. This suggests that fine-tuning diffusion model parameters along with expert-informed data curation can unlock significant gains in model performance. A dual process approach that incorporates expert-informed data curation illustrates both the practicality of involving a single expert for high-volume screening and the value of expert panel-based assessment for dataset refinement. An expert-in-the-loop workflow has the inherent risk of incorporating subjectiveness into the development process [29]. This raises an important question about how differences in clinical judgment may influence model development [11,29].

The addition of quantitative evaluation using FID and KID strengthens these findings by providing an objective complement to subjective expert evaluation which identified Model #3 as the top performing model [24,30]. Across all evaluation subsets, Model #3 consistently achieved the lowest FID and KID values, significantly outperforming both Model #1 and Model #2. Model #2 demonstrated intermediate performance, consistently better than Model #1 in FID, although less so in KID. All KID values were numerically small and close to zero, this is expected because KID estimates the maximum mean discrepancy between two distributions, and the expected value between two samples from the same distribution is zero. In this context, the relative differences between models are more informative than the absolute magnitude. Model #3 consistently showed lower KID values than Models #1 and #2, supporting the FID results and expert grading. Certain KID had overlapping confidence intervals in some small subsets (e.g., Random40) indicated nonsignificant differences. These results confirm that both expert-informed dataset refinement and architectural modifications contributed to measurable gains in performance.

**Figure 3:**
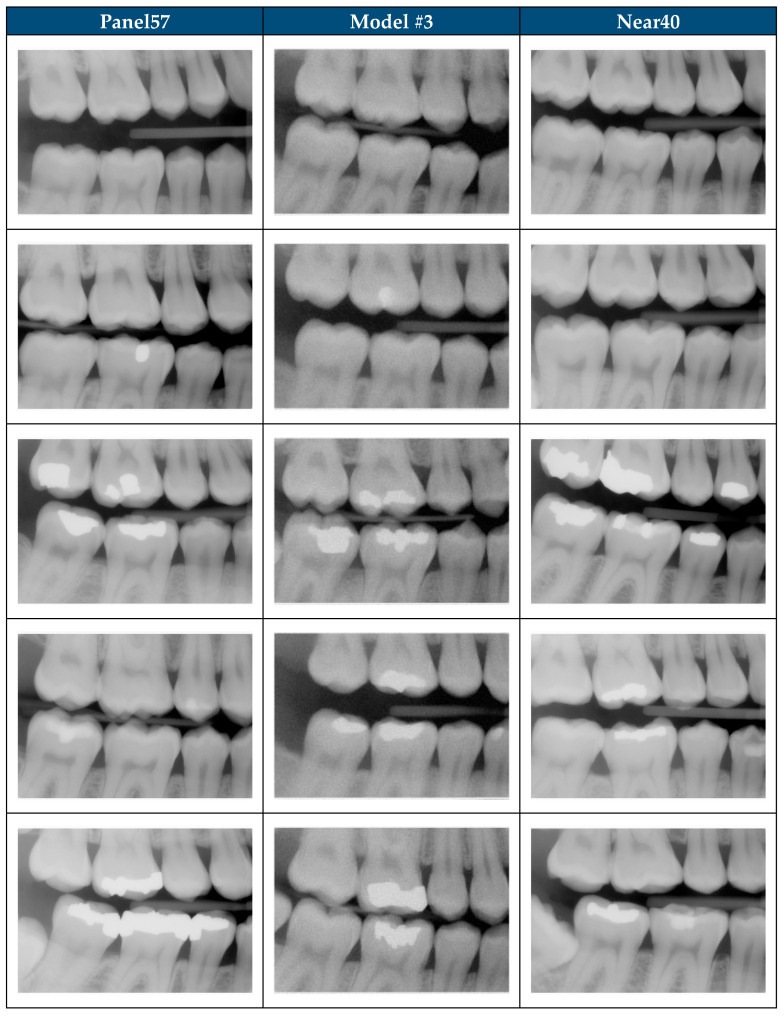
Real and synthetic images from Model #3 FID analysis. In-distribution subset (Panel57) used to develop Model #3. Out-of-distribution subset (Near40) not seen during Model #3 training.

The rank order of performance remained stable across all in-distribution subsets (All200, Panel57, Unseen143) and out-of-distribution subsets (Shift1000, Near40, Random40), with Model #3 demonstrating fidelity the closest to real radiographs in every case (Figure 3). The ΔFID mean was 54.3 when calculated for all comparison subsets between Model #3 and real-vs-real “floor”. This demonstrates that SDRs generated by Model #3 remain distinguishable from real dental radiographs based on FID analysis [31]. A limitation of FID and KID analysis is the feature extractions are performed by the InceptionV3 model [21,32]. The InceptionV3 model was developed using natural images, which may not optimally capture domain-specific features such as enamel–dentin boundaries, periodontal ligament space, or subtle radiographic characteristics [32]. The FID metric is widely used to objectively assess generative model performance but developing radiograph-specific evaluation metrics is necessary to objectively assess fidelity of synthetic medical images [21,32]. These findings demonstrate opportunities for future work to address the limitations of the current diffusion models and explore alternatives to the InceptionV3 model as a backbone for objective metrics like FID and KID [33,34]. The inherent complexity of radiographs underscores the need for domain-specific objective metrics to assess performance of generative models to produce high fidelity synthetic radiographs [24,35].

Clinically, these results provide reassurance that the objective measures and expert ratings converge on a consistent conclusion: Model #3 produces dental radiographs that are both more statistically aligned with real distributions and more likely to be judged as realistic by dentists. This alignment suggests several practical applications. SDRs may be useful for data augmentation, particularly for training diagnostic models in scenarios where rare findings are underrepresented. They may also provide value in simulation-based education, enabling dental students to practice interpretation on large, varied datasets that would be impractical to assemble manually. In addition, SDRs could support methodological development in dental AI, such as pretraining pipelines or benchmarking new diagnostic algorithms.

Building on the current use of an expert panel for training data refinement, subsequent studies will include multiple dentists scoring SDRs, allowing for assessment of inter-rater variability to quantify differences in clinical judgments of realism and clinical adequacy. The subjectivity of radiographic interpretation is not well studied in the context of development of clinically relevant AI models which is critical to developing safe and explainable clinical AI systems. Furthermore, directly assessing the utility of these SDR in downstream AI tasks, such as training image classification or segmentation models, will be crucial for demonstrating their practical value [1]. The algorithm used in Model #3 can be trained with a more robust dataset to expand clinical diversity, then used to study various methods for inclusion of SDRs in training datasets during development of diagnostic models in areas such as caries detection, endodontic pathology, or bone loss assessment. Expanding training data size and robustness, increasing SDR resolution and developing dental domain–specific evaluation metrics, will be key steps toward realizing the full clinical utility of SDR.

Several limitations should be acknowledged. First, the study relied on a relatively small number of curated training images, and the degree to which findings generalize to larger, more diverse populations is unknown. Second, while subsets such as Panel57 and Unseen143 enabled targeted in-distribution evaluation, small-sample groups like Random40 introduced higher variance, and KID differences in these cases did not always achieve statistical significance. Third, FID and KID may underestimate clinically meaningful differences because they are computed in a feature space optimized for natural images [21]. Finally, the standard resolution of a dental radiograph is 100 × 100 micron pixel size and for high-resolution images the pixel size is 75 × 75 microns; the smaller the pixel size the higher the image resolution [36]. A limitation of this study is the dental radiograph resolution was described based on number of pixels (i.e., 128 × 128 pixels) not by pixel size so a direct comparison to the resolution of dental radiographs captured clinically is not possible. Additional work is necessary to achieve a resolution on par with real dental radiographs as lower resolution images limit the visibility of fine anatomical details that may be critical for clinical interpretation [36]. This study provides compelling evidence of diffusion image generation model capability to generate realistic SDRs from a limited amount of real dental radiographs even though continued refinements are necessary to generate higher-resolution images with a pixel size of 75 × 75 microns. Model #3 architecture trained on larger robust dental radiograph datasets has the potential of being a domain-specific foundational model capable of generating very large databases of SDRs.

## 5. Conclusions

This study demonstrates that diffusion models can generate realistic SDRs even from limited training datasets through expert-informed curation and architectural refinement. A single dentist conducted large-scale screening, while a panel of dentists refined the training dataset that guided model development. The progression from Model #1 to Model #3 highlights the importance of both expert-informed data curation and technical optimization in improving SDR realism and fidelity. Objective evaluation using FID and KID confirmed that Model #3 consistently outperformed Models #1 and #2 across all evaluation subsets, while Model #2 showed intermediate performance and Model #1 performed worst. The convergence of subjective expert grading and objective quantitative metrics strengthens confidence in the architecture of Model #3 as a promising domain-specific model to generate SDR databases [24].

## Figures and Tables

**Table 1:** Image Selection Criteria.

Category	Criteria
**Inclusion**	Contain all teeth present distal to 1st premolar to distal of 2nd molar on both maxillary and mandibular arch on the right side.
	The entire crown of each tooth is visible.
	Alveolar crest is visible interproximal between teeth.
**Exclusion**	Unable to identify tooth anatomy.
	Contain any edentulous spaces.
	Poor image quality that requires retake of the image.
	The extent of overlap of proximal contacts requires retake of the image.
	Excessive occlusal plane rotation requires retake of the image.

**Table 2:** Synthetic Dental Radiograph Scoring Rubric.

Category	Description	Sample Images	
**1**	The image appears to be a realistic dental radiograph representative of the training data. (Looks real with no anatomic anomalies)	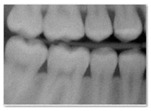	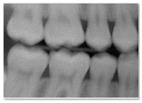
**2**	Image resembles a realistic dental radiograph representative of the training data but contains anatomical hallucinations or abnormalities. (Looks real but tooth count, order or anatomy is unreal)	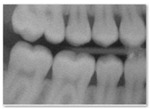	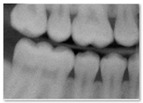
**3**	Image is unrealistic but resembles the general appearance of dental radiograph represented in the training data. (Looks like a dental radiograph with features that are obviously fake)	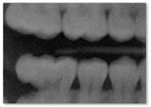	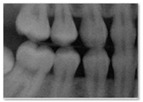
**4**	The image is unrealistic, but portions of the image contain dental-related attributes. (At minimum portions of tooth anatomy are present)	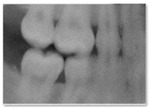	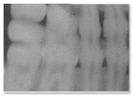
**5**	No recognizable dental-related attributes.	^ 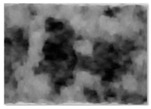 ^	^ 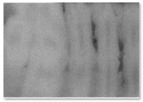 ^

**Table 3:** Real dental radiographs evaluation subsets for FID and KID analysis.

Subset	Total Images	Selection Method	Training Exposure
**All200**	200	BWRM radiographs that met image selection criteria	Model #1: all 200. Models #2–#3: Panel57 only
**Panel57**	57	Expert-panel selected subset of All200	Seen by all 3 models
**Unseen143**	143	Remainder of All200 not in Panel57	Unseen by Model #2 and Model #3
**Shift1000**	1000	Clinically acceptable BWRM excluded from model training	No exposure (all 3 models)
**Near40**	40	Algorithmic-matched subset of Shift1000 (closest to All200)	No exposure (all 3 models)
**Random40 (×5)**	40	Five independent random subsets from Shift1000	No exposure (all 3 models)

**Table 4:** Dental radiograph dataset processing and labeling for model development.

Dataset Description	Number
**Dental Radiographs (unprocessed)**	
Total	10,000
**BWRM subset**	
Total (EP0)	2226
Clinically Acceptable (EP0)	1284
Image Selection Criteria satisfied (EP0)	225
**Expert-Informed Curation subset**	
Total Evaluated (EP)	100
Include (EP)	57
Exclude (EP)	43
**EP Agreement (Image Selection Criteria)**	
Include (4 of 4 EP members)	36
Include (3 of 4 EP members)	7
Include (2 of 4 EP members)	4
Include (1 of 4 EP members)	10
Exclude (4 of 4 EP members)	43

**Table 5:** Model development and impact of refinement on model performance.

Description	Model #1	Model #1	Model #1	Model #2	Model #3
**Training Dataset**					
Total number of images	200	200	200	200	200
Number of unique images	200	200	200	57	57
**Model Training**					
Epochs	22,000	30,000	40,000	30,000	30,000
Diffusion steps	300	300	300	300	600
**Model Performance**					
Total SDR Graded	500	500	500	500	500
Number of Images Scored 1	28	74	9	110	451
Number of Images Scored 2	120	51	34	109	42
Number of Images Scored 3	88	146	35	134	3
Number of Images Scored 4	263	214	379	139	4
Number of Images Scored 5	1	15	43	8	0
Average Score	3.18	3.09	3.83	2.65	1.12
Standard Deviation	0.98	1.11	0.75	1.15	<0.01
Realistic SDR Generation rate	6%	15%	2%	22%	90%
**Model Refinements**					
Refinement	Training Duration (Epochs)	Training Duration (Epochs)	Training Duration (Epochs)	Expert Panel Refined Dataset	Addition of 300 Diffusion Steps
Impact of Refinement on model performance (*p*-Value)	Worse (<0.05)	Improved (<0.05)	Worse (<0.05)	Improved (<0.05)	Improved (<0.05)

**Table 6:** Model-vs-Real comparative analysis reporting FID mean, KID mean and baseline.

Analysis by Subset	Model #1	Model #2	Model #3
**All200**			
FID Real vs. Real “floor” (95% CI)	43.6 (42.2–45.8)		
FID Mean (95% CI)	221.1 (206.9–235.0)	182.2 (176.0–189.3)	114.413 (108.5–120.2)
KID Mean (95% CI)	0.0037 (0.0034–0.0040)	0.0036 (0.0034–0.0039)	0.0024 (0.0021–0.0027)
**Panel57**			
FID Real vs. Real “floor” (95% CI)	80.0 (75.8–84.5)		
FID Mean (95% CI)	233.6 (218.9–250.4)	195.6 (182.9–204.8)	127.3 (118.0–136.8)
KID Mean (95% CI)	0.0038 (0.0035–0.0042)	0.0037 (0.0033–0.0040)	0.0024 (0.0019–0.0028)
**Unseen143**			
FID Real vs. Real “floor” (95% CI)	50.6 (48.5–53.2)		
FID Mean (95% CI)	223.7 (214.4–234.5)	184.0 (176.5–194.4)	117.3 (107.2–129.6)
KID Mean (95% CI)	0.0037 (0.0035–0.0039)	0.0036 (0.0033–0.0039)	0.0024 (0.0020–0.0030)
**Shift1000**			
FID Real vs. Real “floor” (95% CI)	24.5 (24.0–25.1)		
FID Mean (95% CI)	208.2 (200.4–217.2)	174.3 (168.7–179.4)	126.2 (119.1–133.9)
KID Mean (95% CI)	0.0032 (0.0030–0.0036)	0.0033 (0.0030–0.0035)	0.0025 (0.0022–0.0028)
**Near40**			
FID Real vs. Real “floor” (95% CI)	53.1 (50.4–56.4)		
FID Mean (95% CI)	231.2 (213.1–248.2)	197.8 (183.9–209.2)	129.7 (117.0–144.1)
KID Mean (95% CI)	0.0038 (0.0035–0.0042)	0.0039 (0.0035–0.0043)	0.0026 (0.0020–0.0034)
**Random40_1**			
FID Real vs. Real “floor” (95% CI)	107.1 (99.8–117.6)		
FID Mean (95% CI)	239.7 (223.3–253.7)	204.2 (194.2–213.8)	145.7 (134.3–159.2)
KID Mean (95% CI)	0.0034 (0.0031–0.0037)	0.0034 (0.0030–0.0039)	0.0024 (0.0019–0.0032)
**Random40_2**			
FID Real vs. Real “floor” (95% CI)	115.5 (108.6–124.2)		
FID Mean (95% CI)	241.0 (226.9–253.5)	204.8 (196.8–213.6)	161.9 (150.6–174.4)
KID Mean (95% CI)	0.0034 (0.0031–0.0037)	0.0034 (0.0030–0.0039)	0.0028 (0.0023–0.0035)
**Random40_3**			
FID Real vs. Real “floor” (95% CI)	121.5 (115.2–129.3)		
FID Mean (95% CI)	236.3 (220.1–250.8)	200.8 (191.6–209.0)	149.9 (139.6–162.8)
KID Mean (95% CI)	0.0033 (0.0030–0.0036)	0.0033 (0.0029–0.0037)	0.0024 (0.0019–0.0032)
**Random40_4**			
FID Real vs. Real “floor” (95% CI)	112.4 (105.7–123.2)		
FID Mean (95% CI)	228.3 (212.0–244.6)	194.3 (184.9–203.9)	143.4 (132.9–155.4)
KID Mean (95% CI)	0.0030 (0.0027–0.0033)	0.0030 (0.0026–0.0035)	0.0023 (0.0018–0.0031)
**Random40_5**			
FID Real vs. Real “floor” (95% CI)	115.9 (108.1–126.2)		
FID Mean (95% CI)	232.8 (218.1–248.2)	195.9 (187.4–204.7)	151.9 (142.2–162.5)
KID Mean (95% CI)	0.0033 (0.0030–0.0036)	0.0031 (0.0028–0.0036)	0.0024 (0.0020–0.0031)

**Table 7:** Three model Pairwise Δ Analysis.

Subset	Δ FID Mean * (95% CI)		
	**Model #1–Model #2**	**Model #2–Model #3**	**Model #3–Model #1**
All200	38.9 (25.9–55.8)	67.8 (58.6–79.0)	−106.6 (−118.4–−92.0)
Panel57	38.0 (19.5–56.1)	68.3 (55.0–82.6)	−106.4 (−125.3–−90.2)
Unseen143	39.7 (24.4–53.7)	66.7 (52.9–77.1)	−106.4 (−119.7–−89.0)
Shift1000	34.0 (25.6–44.3)	48.1 (40.4–54.2)	−82.1 (−92.9–−71.8)
Near40	33.3 (13.1–54.2)	68.2 (53.3–87.7)	−101.5 (−120.0–−78.0)
Random40_1	35.5 (16.2–52.8)	58.5 (43.4 –75.0)	−94.1 (−112.3–−73.3)
Random40_2	36.1 (18.8–51.3)	43.0 (27.4–59.1)	−79.1 (−98.7–−60.0)
Random40_3	35.5 (15.8–52.9)	50.9 (36.0–66.4)	−86.4 (−104.8–−65.5)
Random40_4	34.0 (15.1–52.7)	51.0 (37.1–66.3)	−85.0 (−102.9–−64.7)
Random40_5	36.9 (20.6–52.4)	44.0 (31.1–59.0)	−81.0 (−101.0–−60.3)

* Δ FID mean = FID [Model A] mean − FID [Model B] mean. If positive Δ FID value, then Model B outperforms Model A. If negative Δ FID value, then Model A outperforms Model B. If 95% CI excludes 0 then Δ FID value is significant.

**Table 8:** Model performance summary of pairwise Δ FID analysis across all subsets.

Evaluation Subset	Lowest FID (Best)	Rank Order
In-distribution Comparison		
All200	Model #3	Model #3 > Model #2 > Model #1
Panel57	Model #3	Model #3 > Model #2 > Model #1
Unseen143	Model #3	Model #3 > Model #2 > Model #1
Out-of-Distribution Comparison		
Shift1000	Model #3	Model #3 > Model #2 > Model #1
Near40	Model #3	Model #3 > Model #2 > Model #1
Random40_1	Model #3	Model #3 > Model #2 > Model #1
Random40_2	Model #3	Model #3 > Model #2 > Model #1
Random40_3	Model #3	Model #3 > Model #2 > Model #1
Random40_4	Model #3	Model #3 > Model #2 > Model #1
Random40_5	Model #3	Model #3 > Model #2 > Model #1

## Data Availability

The data presented in this study are available on request from the corresponding author due to legal restrictions that require appropriate data-sharing agreements or collaborative research and development agreement (CRADA) with the U.S. Army Institute of Surgical Research.

## Abbreviations

The following abbreviations are used in this manuscript:

A2I2	U.S. Army Artificial Intelligence Institute
ADAM	Adaptive Moment Estimation
AI	Artificial Intelligence
BWRM	Bitewing Right Molar
DHA	Defense Health Agency
DICOM	Digital Imaging and Communication in Medicine
ECIA	Enterprise Clinical Image Archive
EP	Expert Panel
FID	Fréchet Inception Distance
GAN	Generative Adversarial Network
GELU	Gaussian Error Linear Unit
GPU	Graphic Processing Unit
JPEG	Joint Photographic Experts Group
KID	Kernel Inception Distance
MMD	Minimum Mean Discrepancy
MSE	Mean Squared Error
SDR	Synthetic Dental Radiograph
SiLU	Sigmoid Linear Unit
SME	Subject Matter Expert
USAISR	U.S. Army Institute of Surgical Research
UIC	Unique Identifier Code
VAE	Variational Autoencoder
